# New Coleoptera records from New Brunswick, Canada: Tenebrionidae and Zopheridae

**DOI:** 10.3897/zookeys.179.2465

**Published:** 2012-04-04

**Authors:** Reginald P. Webster, Jon D. Sweeney, Ian DeMerchant, Patrice Bouchard, Yves Bousquet

**Affiliations:** 1 Natural Resources Canada, Canadian Forest Service - Atlantic Forestry Centre, 1350 Regent St., P.O. Box 4000, Fredericton, NB, Canada E3B 5P7; 2 Canadian National Collection of Insects, Arachnids and Nematodes, Agriculture and Agri-Food Canada, 960 Carling Avenue, Ottawa, Ontario, K1A 0C6, Canada

**Keywords:** Tenebrionidae, Zopheridae, new records, Canada, New Brunswick

## Abstract

Thirteen species of Tenebrionidae are newly reported for New Brunswick, Canada. *Paratenetus punctatus* Spinola, *Pseudocistela brevis* (Say), *Mycetochara foveata* (LeConte), and *Xylopinus aenescens* LeConte are recorded for the first time from the Maritime provinces. *Platydema excavatum* (Say) is removed from the faunal list of New Brunswick, and the presence of *Platydema americanum* Laporte and Brullé for the province is confirmed. This brings the total number of species of Tenebrionidae known from New Brunswick to 42. Two species of Zopheridae, *Bitoma crenata* Fabricius and *Synchita fuliginosa* Melsheimer, are newly recorded for New Brunswick, bringing the number of species known from the province to four. *Bitoma crenata* is new to the Maritime provinces. Collection and habitat data are presented for these species.

## Introduction

The Tenebrionidae is the sixth largest families of beetles, with 1184 species known from North America (Marske and Ivie 2003). Most species occur in arid regions of the southwestern United States, and only 141 species were recorded from Canada by Bousquet and Campbell (1991) and Campbell (1991a). Forty-eight species were reported from the Maritime provinces (Majka et al. 2008). There have been few recent revisions of genera that occur in Canada (*Hymenochara* - Campbell (1978b); *Mycetochara* - Campbell (1978a)), and some members of this family can be difficult to determine to species.

Most Tenebrionidae occurring in eastern Canada are associated with forests and live in or are associated with dead wood; a few are subcortical. A number of species are associated with fruiting bodies of polypore fungi, and a few species are found on flowers and leaves. Most of our adventive species are stored product pests. For more details, see Aalbu et al. (2002).

Majka et al. (2008) reported 33 species of Tenebrionidae from New Brunswick, 13 as new to the province, in their review of the Tenebrionidae of the Maritime provinces (New Brunswick, Nova Scotia, Prince Edward Island). Here, we report 13 additional species for the province.

The Zopheridae, which includes the Colydiidae (Ślipiński and Lawrence 1999), contains only a few species in eastern Canada. Adults live under bark or in rotten wood, and some species feed on fruiting bodies of polypore fungi (Phellopsini) (Ślipiński and Lawrence 1999; Ivie 2002). See Ivie (2002) for a general review of the North American members of this family. Three species (*Phellopsis obcordata* (Kirby), *Lasconotus borealis* Horn, *Synchita fuliginosa* Melsheimer) were reported for the Maritime provinces (Bousquet 1991; Campbell 1991b; Majka et al. 2006). Only *Lasconotus borealis* and *Phellopsis obcordata* were reported from New Brunswick (Bousquet 1991; Majka et al. 2006; Foley and Ivie 2008). Here, we report two additional species for the province.

## Methods and conventions

The following records are based on specimens collected during a general survey by the first author to document the Coleoptera fauna of New Brunswick and from by-catch samples obtained during a study to develop a general attractant for the detection of invasive species of Cerambycidae.

### Collection methods

Various collection methods were employed to collect the species reported in this study. Details are outlined in Campbell (1973) and Webster et al. (2009, Appendix). See Webster et al. (in press) for details of the methods used for deployment of Lindgren 12-funnel traps and sample collection. A description of the habitat was recorded for all specimens collected during this survey. Locality and habitat data are presented exactly as on labels for each record. This information, as well as additional collecting notes, is summarized and discussed in the collection and habitat data section for each species.

### Distribution

Distribution maps, created using ArcMap and ArcGIS, are presented for each species in New Brunswick. Every species is cited with current distribution in Canada and Alaska, using abbreviations for the state, provinces, and territories. New records for New Brunswick are indicated in bold under Distribution in Canada and Alaska. The following abbreviations are used in the text:

**Table:** 

**AK**	Alaska	**MB**	Manitoba
**YT**	Yukon Territory	**ON**	Ontario
**NT**	Northwest Territories	**QC**	Quebec
**NU**	Nunavut	**NB**	New Brunswick
**BC**	British Columbia	**PE**	Prince Edward Island
**AB**	Alberta	**NS**	Nova Scotia
**SK**	Saskatchewan	**NF & LB**	Newfoundland and Labrador*

*Newfoundland and Labrador are each treated separately under the current Distribution in Canada and Alaska.

Acronyms of collections examined or where specimens reside referred to in this study are as follows:

**AFC** Atlantic Forestry Centre, Natural Resources Canada, Canadian Forest Service, Fredericton, New Brunswick, Canada

**CNC** Canadian National Collection of Insects, Arachnids and Nematodes, Ottawa, Ontario, Canada

**NBM** New Brunswick Museum, Saint John, New Brunswick, Canada

**RWC** Reginald P. Webster Collection, Charters Settlement, New Brunswick, Canada

## Results

Thirteen species of Tenebrionidae are newly reported for New Brunswick. *Paratenetus punctatus* Spinola, *Pseudocistela brevis* (Say), *Mycetochara foveata* (LeConte), and *Xylopinus aenescens* LeConte are recorded from the Maritime provinces for the first time; *Platydema excavatum* (Say) is removed from the faunal list of New Brunswick, and the presence of *Platydema americanum* in New Brunswick is confirmed. This brings the total number of species known from New Brunswick to 42 (Table 1).

**Table 1.** Species of Tenebrionidae and Zopheridae recorded from New Brunswick, Canada.

**Family Tenebrionidae Latreille**
**Subfamily Lagriinae Latreille**
**Tribe Lagriini Latreille**
*Arthromacra aenea* (Say)
**Tribe Goniaderini Lacodaire**
*Paratenetus punctatus* Spinola**
*Paratenetus* (undescribed species)
**Subfamily Tenebrioninae Latreille**
**Tribe Alphitobiini Reitter**
*Alphitobius diaperinus* (Panzer)
**Tribe Bolitophagini Kirby**
*Bolitophagus corticola* Say
*Bolitotherus cornutus* (Panzer)
*Eleates depressus* (Randall)
**Tribe Helopini Latreille**
*Helops gracilis* Bland
**Tribe Opatrini Brullé**
*Blapstinus metallicus* (Fabricius)
**Tribe Tenebrionini Latreille**
*Neatus tenebrioides* (Palisot de Beauvois)
*Neatus* (undescribed species)
*Tenebrio molitor* Linnaeus
**Tribe Triboliini Gistel**
*Latheticus oryzae* Waterhouse
*Tribolium audax* Halstead
*Tribolium castaneum* (Herbst)*
*Tribolium destructor* Uyttenboogart
*Tribolium madens* (Charpentier)
**Subfamily Alleculinae Laporte**
**Tribe Alleculini Laporte**
*Androchirus erythropus* (Kirby)
*Capnochroa fuliginosa* (Melsheimer)
*Pseudocistela brevis* (Say)**
*Hymenorus molestus* Fall
*Hymenorus niger* (Melsheimer)
*Hymenorus obesus* Casey
*Isomira quadristriata* (Couper)
*Isomira sericea* (Say)*
*Mycetochara analis* (LeConte)*
*Mycetochara bicolor* (Couper)*
*Mycetochara binotata* (Say)*
*Mycetocara fraterna* (Say)
*Mycetochara foveata* (LeConte)**
**Subfamily Diaperinae Latreille**
**Tribe Diaperini Latreille**
*Diaperis maculata* Olivier
*Neomida bicornis* (Fabricius)*
*Platydema americanum* Laporte and Brullé
*Platydema teleops* Triplehorn*
**Tribe Hypophlaeini Billberg**
*Corticeus praetermissus* (Fall)*
*Corticeus tenuis* (LeConte)
**Tribe Scaphidemini Reitter**
*Scaphidema aeneolum* (LeConte)
**Subfamily Stenochiinae Kirby**
**Tribe Cnodalonini Oken**
*Alobates pennsylvanicus* (DeGeer)
*Iphthiminus opacus* (LeConte)
*Upis ceramboides* (Linnaeus)
*Xylopinus aenescens* LeConte**
*Xylopinus saperdioides* (Olivier)*
**Family Zopheridae Solier**
**Subfamily Colydiinae Billberg**
**Tribe Synchitini Erichson**
*Bitoma crenata* Fabricius**
*Lasconotus borealis* Horn
*Synchita fuliginosa* Melsheimer*
**Subfamily Zopherinae Solier**
**Tribe Phellopsini Ślipiński and Lawrence**
*Phellopsis obcordata* (Kirby)

**Notes:** *New to province; **New to Maritime provinces.

### Species Accounts

All records below are species newly recorded for New Brunswick, Canada, unless noted otherwise (additional records). Species followed by ** are newly recorded from the Maritime provinces of Canada.

The classification of the Zopheridae and Tenebrionidae follows Bouchard et al. (2011).

### Family Tenebrionidae Latreille, 1802

**Subfamily Lagriinae Latreille, 1825**

**Tribe Goniaderini Lacodaire, 1859**

#### Paratenetus punctatus

Spinola, 1844**

http://species-id.net/wiki/Paratenetus_punctatus

Map 1

##### Material examined.

**New Brunswick, Carleton Co.**,Jackson Falls,“Bell Forest”, 46.2200°N, 67.7231°W, 28.VI.2005, R. P. Webster, hardwood forest, u.v. light (1, RWC); same locality but 46.2150°N, 67.7190°W, 24.VI.2005, J. Edsall and R. Webster, river margin, sweeping foliage (1, RWC). **Charlotte Co.**, 10 km NW of New River Beach, 45.2110°N, 66.6170°W, 10–26.V.2010, R. Webster & C. MacKay, old growth eastern white cedar forest, Lindgren funnel trap (1, AFC). **Northumberland Co.**, 12 km SSE of Upper Napan, 46.8991°N, 65.3682°W, 7.VI.2006, R. P. Webster, eastern white cedar swamp, in moss and leaf litter (1, RWC). **Queens Co.**, Cranberry Lake P.N.A. (Protected Natural Area), 46.1125°N, 65.6075°W, 25.V.–5.VI.2009, 5–11.VI.2009, R. Webster & M.-A. Giguère, mature red oak forest, Lindgren funnel traps (5, AFC). **Sunbury Co.**, Acadia Research Forest, 45.9866°N, 66.3841°W, 8–13.V.2009, 13–19.V.2009, 19–25.V.2009, 25.V–2.VI.2009, 2–9.VI.2009, 24–30.VI.2009, R. Webster & M.-A. Giguère, mature (110 year-old) red spruce forest with scattered red maple and balsam fir, Lindgren funnel traps (9, AFC, RWC). **York Co.**, Charters Settlement, 45.8267°N, 66.7343°W, 16.IV.2005, R. P. Webster, *Carex* marsh, in litter and sphagnum at base of tree (1, RWC); same locality and collector but 45.8310°N, 66.7340°W, 12.VII.2005, regenerating mixed forest, beating foliage of red pine (1, RWC); same locality and collector but 45.8340°N, 66.7450°W, mixed forest, beating birch branches with dead dried leaves (3, RWC); Canterbury, Browns Mountain Fen, 45.8967°N, 67.6343°W, 2.V.2005, 13.V.2005, R. Webster & M.-A. Giguère, calcareous cedar fen, in moss and litter at base of tree (2, NBM, RWC); 15 km W of Tracy off Rt. 645, 45.6848°N, 66.8821°W, 19–25.V.2009, R. Webster & M.-A. Giguère, old red pine forest, Lindgren funnel trap (1, RWC); 14 km WSW of Tracy, S of Rt. 645, 45.6741°N, 66.8661°W, 25.IV–10.V.2010, 25.V–2.V.2010, R. Webster & C. MacKay, old mixed forest with red and white spruce, red and white pine, balsam fir, eastern white cedar, red maple, and *Populus* sp., Lindgren funnel traps (2, AFC).

**Map 1.**
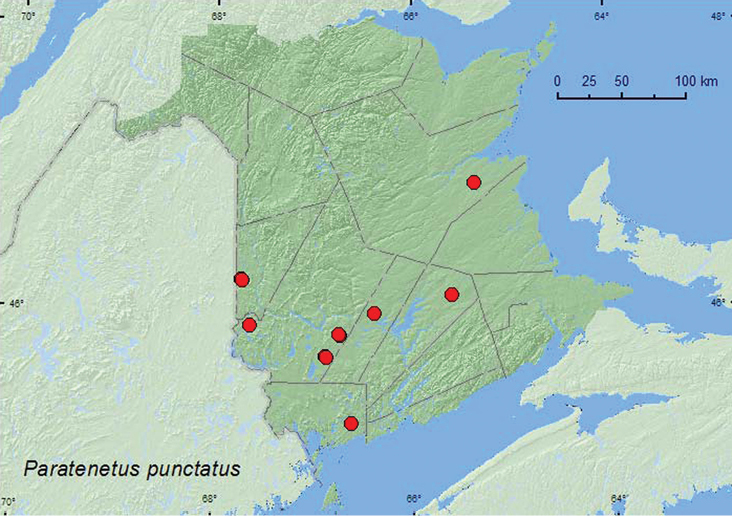
Collection localities in New Brunswick, Canada of *Paratenetus punctatus*.

##### Collection and habitat data.

This species was collected from various forest types in New Brunswick, including hardwood forests with sugar maple (*Acer saccharum* Marsh.) and American beech (*Fagus grandifolia* Ehrh.), a red oak (*Quercus rubra* L.) forest, eastern white cedar (*Thuja occidentalis* L.) forests, an old red pine (*Pinus resinosa* Ait.) forest, and mixed forests. Most adults were collected from Lindgren funnel traps (29). Adults with specific microhabitat data were collected from moss and leaf litter at base of trees, beating or sweeping foliage, and beating birch branches that had dead dried leaves. Adults were collected during April, May, June, and July.

##### Distribution in Canada and Alaska.

ON, QC, **NB** (Campbell 1991a).

### Subfamily Tenebrioninae Latreille, 1802

**Tribe Bolitophagini Kirby, 1837**

#### Eleates depressus

(Randall 1838)

http://species-id.net/wiki/Eleates_depressus

Map 2

##### Material examined.

**Additional New Brunswick record. York Co.**, Charters Settlement, 45.8395°N, 66.7391°W, 25.VI.2009, R. P. Webster, mixed forest, u.v. light (1, RWC).

**Map 2.**
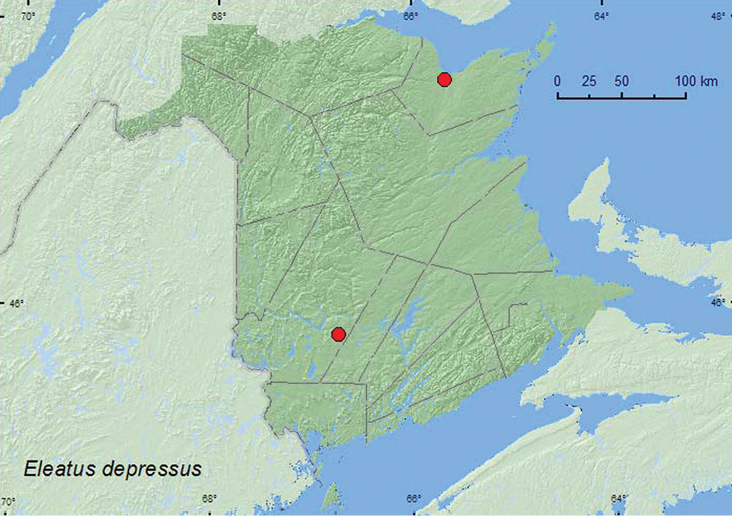
Collection localities in New Brunswick, Canada of *Eleates depressus*.

##### Collection and habitat data.

One specimen was collected at an ultraviolet light in a mixed forest during June. Dearborn and Donahue (1993) reported this species from under bark of pines in Maine.

##### Distribution in Canada and Alaska.

MB, ON, QC, NB (Bousquet and Campbell 1991). The only previous record of this species from New Brunswick was from Bathurst (specimens in CNC).

### Tribe Tenebrionini Latreille, 1802

#### Neatus tenebrioides

(Palisot de Beauvois, 1805)

http://species-id.net/wiki/Neatus_tenebrioides

Fig. 1 Map 3

##### Material examined.

**Additional New Brunswick records. Carleton Co.**,Jackson Falls,“Bell Forest”, 46.2210°N, 67.7210°W, 12.VII.2004, 13.VII.2004, K. Bredin, J. Edsall, & R. Webster, mature hardwood forest, under bark and in u.v. light trap (2, RWC); same locality and forest type, 26.VI.2007, R. P. Webster, on trunk of recently fallen *Tilia americana*, collected at night with aid of headlamp (5, RWC). **Queens Co.**, Cranberry Lake P.N.A., 46.1125°N, 65.6075°W, 15–29.VI.2009, 15–21.VII.2009, R. Webster & M.-A. Giguère, mature red oak forest, Lindgren funnel traps (2, AFC); same locality data but 28.VII.2009, R. Webster & M.-A. Giguère, mature red oak forest, u.v. light. (1, AFC); Grand Lake Meadows P.N.A., 45.8227°N, 66.1209°W, 21.VI–5.VII.2011, M. Roy & V. Webster, old silver maple forest with green ash and seasonally flooded marsh, Lindgren funnel traps in forest canopy (3, AFC, NBM). **Sunbury Co.**, Burton, near Sunpoke Lake, 45.7663°N, 66.5550°W, 20.VII.2006, oak forest, under loose bark of oak (1, RWC). **York Co.**, Fredericton, 14.VIII.1932, R. E. Balch (1, AFC); Fredericton, insectary, 2.VI.1980 (probably reared) (no collector given) (27, AFC);Charters Settlement, 45.8395°N, 66.7391°W, 20.VII.2006, R. P. Webster, mixed forest, u.v. light (1, RWC).

**Figures 1–6.**
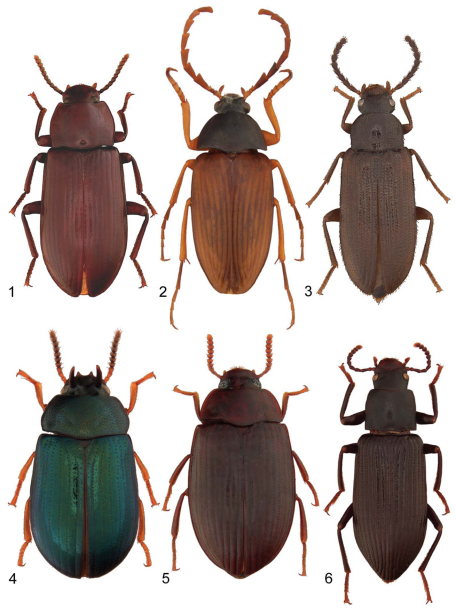
Representative species of the family Tenebrionidae in New Brunswick: **1** *Neatus tenebrioides* (Palisot de Beauvois, 1805) **2** *Pseudocistela brevis* (Say, 1824) **3** *Mycetochara foveata* (LeConte, 1866) **4** *Neomida bicornis* (Fabricius, 1777) **5** *Platydema americanum* Laporte and Brullé, 1831 **6** *Xylopinus saperdioides* (Olivier, 1795).

**Map 3.**
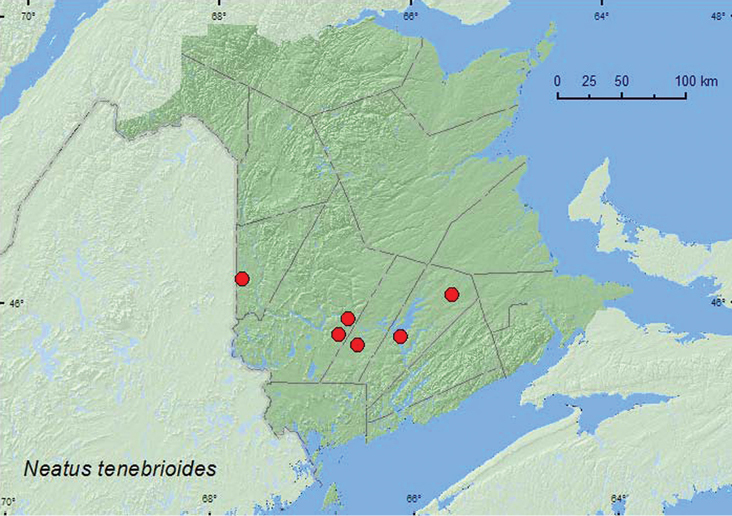
Collection localities in New Brunswick, Canada of *Neatus tenebrioides*.

##### Collection and habitat data.

Adults were collected from under loose bark of a red oak, at an ultraviolet light, on the trunk of recently fallen *Tilia americana* L. at night with the aid of a headlamp, and from Lindgren funnel traps. This species was collected in hardwood (sugar maple and beech, red oak, silver maple) and mixed forests during June, July, and August.

##### Distribution in Canada and Alaska.

BC, MB, ON, QC, NB (Bousquet and Campbell 1991). Considering the number of recent records, it was surprising that this species was known from only one locality (Restigouche Co., Sea Side) in New Brunswick and the Maritime provinces (See Majka et al. 2008).

### Tribe Triboliini Gistel, 1848

#### Tribolium castaneum

(Herbst, 1797)

http://species-id.net/wiki/Tribolium_castaneum

Map 4

##### Material examined.

**New Brunswick, Westmorland Co.**, Moncton, 21.IV.1945, R. S. Forbes (3, AFC).

**Map 4.**
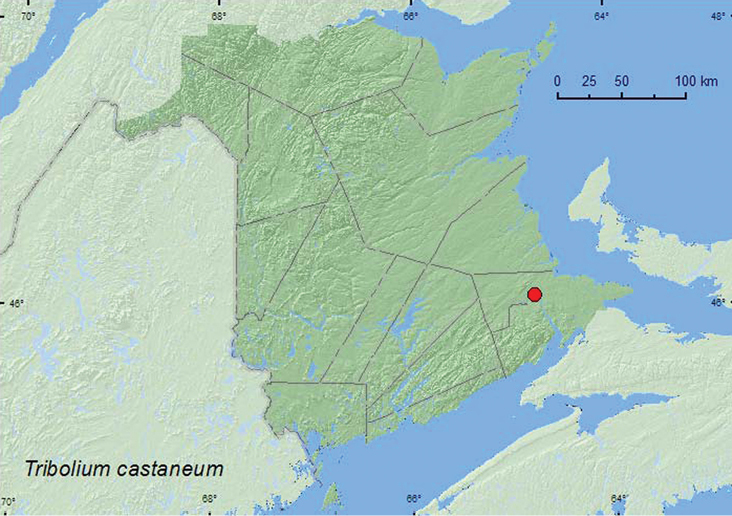
Collection localities in New Brunswick, Canada of *Tribolium castaneum*.

##### Collection and habitat data.

No habitat information was included with the label data.This adventive species is considered a stored grain pest (Bousquet 1990).

##### Distribution in Canada and Alaska.

BC, AB, SK, MB, ON, QC, **NB**, PE, NS (Bousquet and Campbell 1991; Majka et al. 2008).

### Subfamily Alleculinae Laporte, 1840

**Tribe Alleculini Laporte, 1840**

#### Pseudocistela brevis

(Say, 1824)**

http://species-id.net/wiki/Pseudocistela_brevis

Fig. 2 Map 5

##### Material examined.

**New Brunswick, Queens Co.**, Cranberry Lake P.N.A., 46.1125°N, 65.6075°W, 10–15.VII.2009, R. Webster & M.-A. Giguère, old red oak forest, Lindgren funnel trap (1, RWC); same locality data and forest type, 29.VI–7.VII.2011, 13–20.VII.2011, M. Roy & V. Webster, Lindgren funnel traps in forest canopy (4, AFC, NBM, RWC).

**Map 5.**
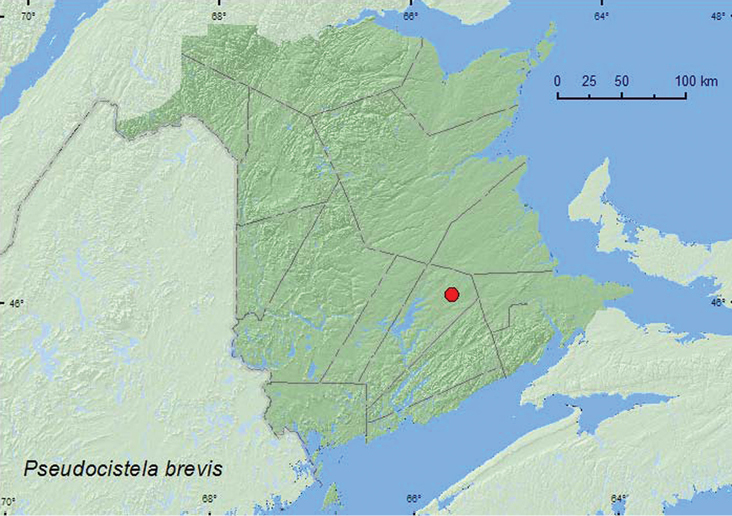
Collection localities in New Brunswick, Canada of *Pseudocistela brevis*.

##### Collection and habitat data.

Mostadults from New Brunswick were captured during July in Lindgren funnel traps deployed in the forest canopy of a red oak forest.

##### Distribution in Canada and Alaska.

ON, QC, **NB** (Bousquet and Campbell 1991).

#### Isomira sericea

(Say, 1824)

http://species-id.net/wiki/Isomira_sericea

Map 6

##### Material examined.

**New Brunswick, Northumberland Co.**, Blueberry Rd. off Hwy 8, 47.3210°N, 65.4228°W, 24.VII.2005, R. P. Webster, jack pine forest, on foliage of jack pine (1, RWC).

**Map 6.**
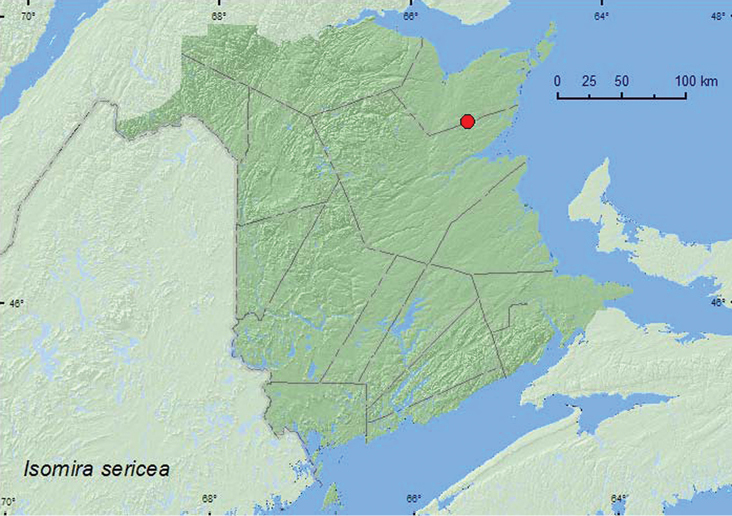
Collection localities in New Brunswick, Canada of *Isomira sericea*.

##### Collection and habitat data.

The single New Brunswick specimen was beaten from foliage of jack pine (*Pinus banksiana* Lamb.) in a jack pine forest. Majka et al. (2008) reported it from southern Nova Scotia from mixed forests, coastal barrens, and jack pine forests, and on flowers of Virginia rose (*Rosa virginiana* Mill.) and bush honeysuckle (*Diervilla lonicera* P. Mill.).

##### Distribution in Canada and Alaska.

ON, QC, **NB**, NS (Bousquet and Campbell 1991).

#### Mycetochara analis

(LeConte, 1878)

http://species-id.net/wiki/Mycetochara_analis

Map 7

##### Material examined.

**New Brunswick, Queens Co.**, Grand Lake Meadows P.N.A., 45.8227°N, 66.1209°W, 31.V–15.VI.2010, R. Webster & C. MacKay, old silver maple forest with green ash (*Fraxinus pennsylvanica* Marsh.) and seasonally flooded marsh, Lindgren funnel trap (1, RWC); same locality data and forest type, 3–21.VI.2011, 21.VI–5.VII.2011, 5–19.VII.2011, M. Roy & V. Webster, Lindgren funnel traps (11, AFC, NBM, RWC).

**Map 7.**
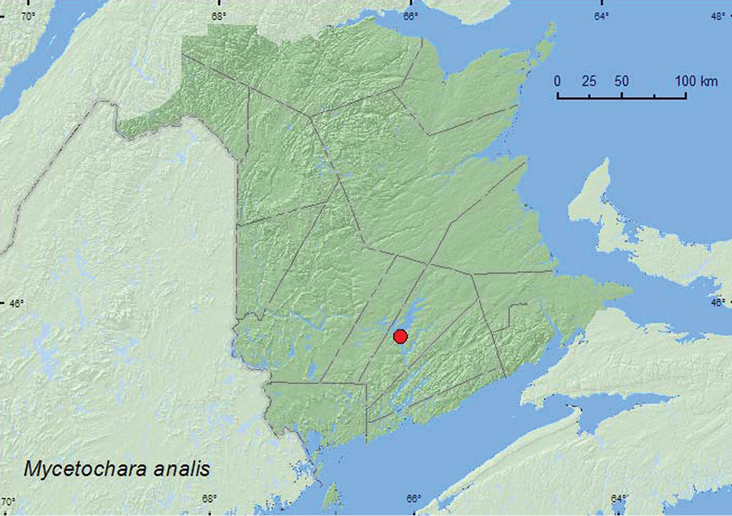
Collection localities in New Brunswick, Canada of *Mycetochara analis*.

##### Collection and habitat data.

All specimens werecaptured during June and July in Lindgren funnel traps deployed in an old silver maple (*Acer saccharinum* L.) swamp.

##### Distribution in Canada and Alaska.

BC, SK, MB, ON, QC, **NB**, NS (Bousquet and Campbell 1991; Bishop et al. 2009).

#### Mycetochara bicolor

(Couper, 1865)

http://species-id.net/wiki/Mycetochara_bicolor

Map 8

##### Material examined.

**New Brunswick, Carleton Co.**, Jackson Falls, “Bell Forest”, 46.2200°N, 67.7231°W, 13.VII.2004, K. Bredin, J. Edsall, & R. Webster, mature hardwood forest, u.v. light (1, RWC); same locality and forest type, 12–19.VI.2008, 19–27.VI.2008, 27.VI–5.VII.2008, 5–12.VII.2008, 12–19.VII.2008, R. P. Webster, Lindgren funnel traps (6, AFC, RWC). **Charlotte Co.**, 10 km NW of New River Beach, 45.2110°N, 66.6170°W, 29.VI–16.VII.2010, R. Webster & C. MacKay, old growth eastern white cedar forest, Lindgren funnel trap (1, AFC). **Queens Co.**, Cranberry Lake P.N.A., 46.1125°N, 65.6075°W, 1–10.VII.2009, R. Webster & M.-A. Giguère, mature red oak forest, Lindgren funnel trap (1, RWC); Grand Lake Meadows P.N.A., 45.8227°N, 66.1209°W, 15–29.VI.2010, R. Webster & C. MacKay, old silver maple forest with green ash and seasonally flooded marsh, Lindgren funnel traps (5, AFC); same locality data and forest type, 21.VI–5.VII.2011, M. Roy & V. Webster, Lindgren funnel traps (3, AFC, NBM). **Restigouche Co.**, Dionne Brook P.N.A., 47.9030°N, 68.3503°W, 28.VII–9.VIII.2011, M. Roy & V. Webster, old-growth northern hardwood forest, Lindgren funnel trap (1, NBM). **Sunbury Co.**, Acadia Research Forest, 45.9866°N, 66.3841°W, 16–24.VI.2009, 8–13.VII.2009, 13–21.VII.2009, R. Webster & M.-A. Giguère, mature (110 year-old) red spruce forest with scattered red maple and balsam fir, Lindgren funnel traps (4, AFC, RWC). **York Co.**, 15 km W of Tracy off Rt. 645, 45.6848°N, 66.8821°W, 28.VI–7.VII.2009, 7–14.VII.2009, R. Webster & M.-A. Giguère, old red pine forest, Lindgren funnel traps (3, AFC, RWC); same locality and forest type but, 7–14.VII.2010, R. Webster & C. MacKay, Lindgren funnel trap (1, AFC); 14 km WSW of Tracy, S of Rt. 645, 45.6741°N, 66.8661°W, 16–30.VI.2010, R. Webster and C. MacKay, old mixed forest with red and white spruce, red and white pine, balsam fir, eastern white cedar, red maple, and *Populus* sp., Lindgren funnel trap (1, AFC).

**Map 8.**
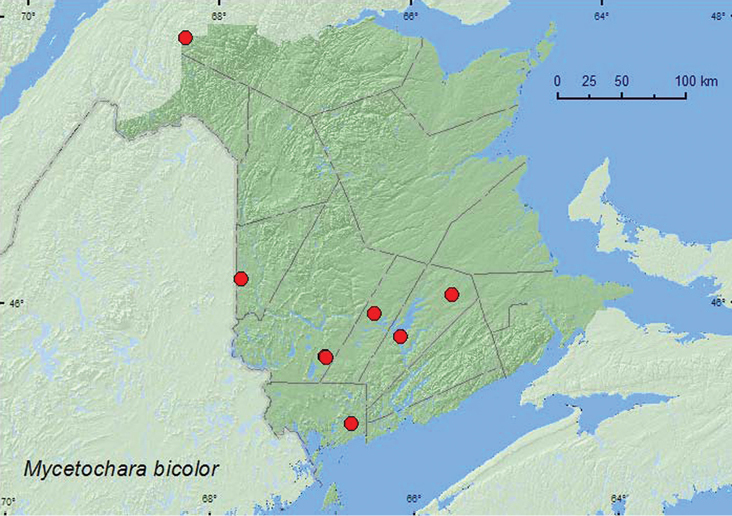
Collection localities in New Brunswick, Canada of *Mycetochara bicolor*.

##### Collection and habitat data.

This species was captured in a hardwood forest (sugar maple and beech), an old-growth northern hardwood forest, an old silver maple forest, an old red oak forest, an old mixed forest, an old red pine forest, a mature red spruce (*Picea rubens* Sarg.) forest, and an old eastern white cedar forest. Most adults were captured in Lindgren funnel traps; a few at an ultraviolet light. Majka et al. (2008) reported this species from under bark of red spruce in Nova Scotia. Adults were captured during June and July.

##### Distribution in Canada and Alaska.

ON, QC, **NB**, NS (Bousquet and Campbell 1991; Majka et al. 2008).

#### Mycetochara binotata

(Say, 1824)

http://species-id.net/wiki/Mycetochara_binotata

Map 9

##### Material examined.

**New Brunswick, Carleton Co.**, Jackson Falls, “Bell Forest”, 46.2200°N, 67.7231°W, 26.VI.2007, 8.VII.2008, R. P. Webster, mature hardwood forest, u.v. light (2, RWC); same locality, collector, and forest type, 5–12.VII.2008, Lindgren funnel trap (1, RWC). **Queens Co.**, Cranberry Lake P.N.A., 46.1125°N, 65.6075°W, 10–15.VII.2009, R. Webster & M.-A. Giguère, mature red oak forest, Lindgren funnel trap (1, RWC); same locality data and forest type, 22–29.VI.2011, M. Roy & V. Webster, Lindgren funnel traps (2, NBM); Grand Lake Meadows P.N.A., 45.8227°N, 66.1209°W, 15–29.VI.2010, 12–26.VII.2010, R. Webster & C. MacKay, old silver maple forest with green ash and seasonally flooded marsh, Lindgren funnel traps (2, NBM, RWC); same locality data and forest type, 21.VI–5.VII.2011, 19.VII–5.VIII.2011, M. Roy & V. Webster, Lindgren funnel traps (6, AFC, NBM, RWC). **Restigouche Co.**, Dionne Brook P.N.A., 47.9030°N, 68.3503°W, 14–28.VII.2011, M. Roy & V. Webster, old-growth northern hardwood forest, Lindgren funnel trap (1, NBM). **Sunbury Co.**, Burton, near Sunpoke Lake, 45.7658°N, 66.5546°W, 29.VII.2007, oak forest, u.v. light (1, NBM); Acadia Research Forest, 45.9866°N, 66.3841°W, 13–21.VII.2009, R. Webster & M.-A. Giguère, mature (110 year-old) red spruce forest with scattered red maple and balsam fir, Lindgren funnel trap (1, RWC). **York Co.**, Charters Settlement, 45.8395°N, 66.7391°W, 9.VII.2006, R. P. Webster, mixed forest, u.v. light (1, RWC); 15 km W of Tracy off Rt. 645, 45.6848°N, 66.8821°W, 28.VI–7.VII.2009, R. Webster & M.-A. Giguère, old red pine forest, Lindgren funnel trap (1, RWC).

**Map 9.**
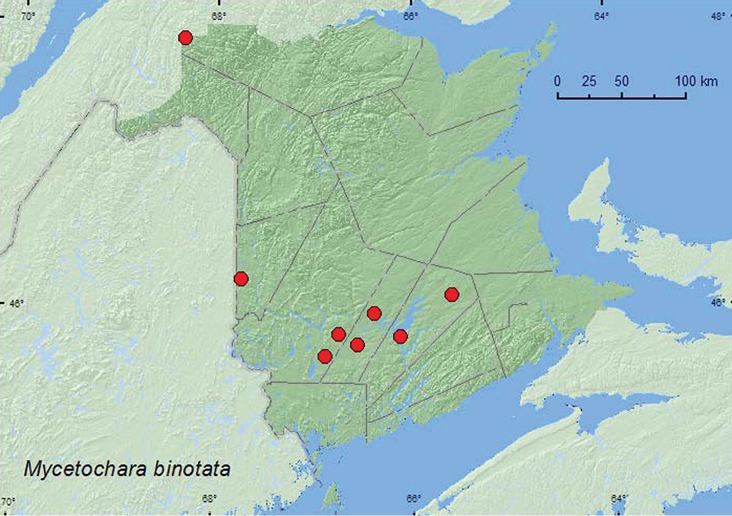
Collection localities in New Brunswick, Canada of *Mycetochara binotata*.

##### Collection and habitat data.

This species was captured in a hardwood forest (sugar maple and beech), an old-growth northern hardwood forest, an old silver maple forest, an old red oak forest, a mixed forest, an old red pine forest, and a mature (110-year-old) red spruce forest. Most adults were captured in Lindgren funnel traps; a few at an ultraviolet light. Adults were captured during June and July.

##### Distribution in Canada and Alaska.

ON, QC, **NB**, NS (Bousquet and Campbell 1991; Majka et al. 2008).

#### Mycetochara foveata

(LeConte, 1866)**

http://species-id.net/wiki/Mycetochara_foveata

Fig. 3 Map 10

##### Material examined.

**New Brunswick, Carleton Co.**, Jackson Falls, “Bell Forest”, 46.2200°N, 67.7231°W, 27.VI–5.VII.2008, 5–12.VII.2008, R. P. Webster, mature hardwood forest, Lindgren funnel traps (3, AFC, RWC). **Queens Co.**, Cranberry Lake P.N.A., 46.1125°N, 65.6075°W, 18–25.VI.2009, R. Webster & M.-A. Giguère, mature red oak forest, Lindgren funnel trap (1, AFC); same locality data and forest type, 29.VI–7.VII.2011, M. Roy & V. Webster, Lindgren funnel traps (2, NBM, RWC); Grand Lake Meadows P.N.A., 45.8227°N, 66.1209°W, 29.VI–12.VII.2010, R. Webster, C. MacKay, M. Laity, & R. Johns, silver maple swamp and seasonally flooded marsh, Lindgren funnel trap in forest canopy (1, AFC); same locality and forest type, 21.VI–5.VII.2011, M. Roy & V. Webster, Lindgren funnel trap (1, NBM). **York Co.**, 15 km W of Tracy off Rt. 645, 45.6848°N, 66.8821°W, 28.VI–7.VII.2009, 7–14.VII.2009, R. Webster & M.-A. Giguère, old red pine forest, Lindgren funnel traps (6, AFC, RWC); same locality and habitat data 16–30.VI.2010, 30.VI–13.VII.2010, R. Webster & C. MacKay, Lindgren funnel traps (3, AFC, RWC).

**Map 10.**
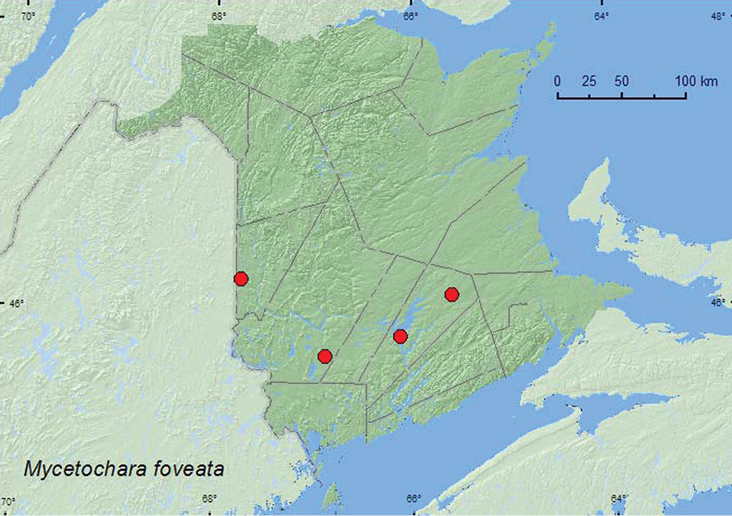
Collection localities in New Brunswick, Canada of *Mycetochara foveata*.

##### Collection and habitat data.

New Brunswick specimens were captured in Lindgren funnel traps deployed in a mature hardwood forest (sugar maple and beech), an old red oak forest, an old silver maple forest, and an old red pine forest. Adults were captured during June and July.

##### Distribution in Canada and Alaska.

ON, QC, **NB** (Bousquet and Campbell 1991).

### Subfamily Diaperinae Latreille, 1802

**Tribe Diaperini Latreille, 1082**

#### Neomida bicornis

(Fabricius, 1777)

http://species-id.net/wiki/Neomida_bicornis

Fig. 4 Map 11

##### Material examined.

**New Brunswick, Carleton Co.**,Jackson Falls,“Bell Forest”, 46.2200°N, 67.7231°W, 9.X.2006, R. P. Webster, mature hardwood forest, under bark of fallen beech log covered with polypore fungi (2, RWC). **Queens Co.**, Cranberry Lake P.N.A., 46.1125°N, 65.6075°W, 12–21.V.2009, R. Webster & M.-A. Giguère, mature red oak forest, Lindgren funnel trap (1, AFC); same locality data but 14.VIII.2009, R. Webster & M.-A. Giguère, margin of red oak forest in bracket fungi on sun-exposed stump (8, AFC, RWC). **York Co.,** Charters Settlement, 45.8395°N, 66.7391°W, 19.IV.2004, R. P. Webster, mixed forest, under bark (2, AFC, RWC).

**Map 11.**
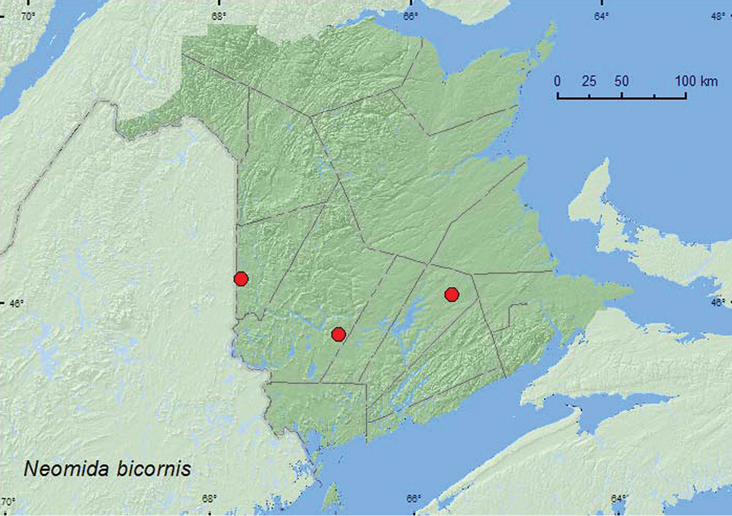
Collection localities in New Brunswick, Canada of *Neomida bicornis*.

##### Collection and habitat data.

*Neomida bicornis* from New Brunswick were collected from under bark, in bracket (polypore) fungi on a sun-exposed stump, and under bark of an American beech log covered with polypore fungi in hardwood and mixed forests. One individual was a victim of a Lindgren funnel trap. Adults were collected during April, May, August, and October. Majka et al. (2008) reported this species from similar habitats in Nova Scotia.

##### Distribution in Canada and Alaska.

ON, QC, **NB**, PE, NS (Bousquet and Campbell 1991; Majka et al. 2008).

#### Platydema americanum

Laporte and Brullé, 1831

http://species-id.net/wiki/Platydema_americanum

Fig. 5 Map 12

##### Material examined.

**Additional New Brunswick records, Carleton Co.**, Hartland, Becaguimec Island, 46.3106°N, 67.5393°W, 13.IX.2006, R. P. Webster, mature mixed forest, in large dried polypore fungi (1, RWC); Two Mile Brook Fen, 46.3702°N, 67.6772°W, 4.VIII.2006, R. P. Webster, mixed forest, in gilled mushroom (1, NBM). **Restigouche Co.,** Jacquet River Gorge P.N.A., 47.8160°N, 66.0083°W, 14.VIII.2010, R. P. Webster, old eastern white cedar forest, in polypore fungi on *Populus* log (3, NBM, RWC). **Sunbury Co.**, 45.9007°N, 66.2423°W. 27.VIII.2006, R. P. Webster, silver maple swamp, among polypore fungi on poplar log (2, RWC). **York Co.,** Charters Settlement, 45.8188°N, 66.7460°W, 28.XI.2004, R. P. Webster, clear-cut, under bark of conifer stump (1, RWC); same locality and collector but 45.8340°N, 66.7450°W, 11.VII.2006, 20.V.2007, mixed forest, on partially dried *Pleurotus* sp. on dead standing trembling aspen (2, RWC); Canterbury, near Browns Mountain Fen, 45.8876°N, 67.6560°W, 3.VIII.2006, R. P. Webster, hardwood forest, in slightly dried *Pleurotus* sp. on sugar maple (1, NBM); NW of Hwy 2 exit 271, 45.8776°N, 66.8254°W, 8.VI.2008, S. Clayden, mixed forest, in (*Pleurotus*) mushrooms on log (1, RWC).

**Map 12.**
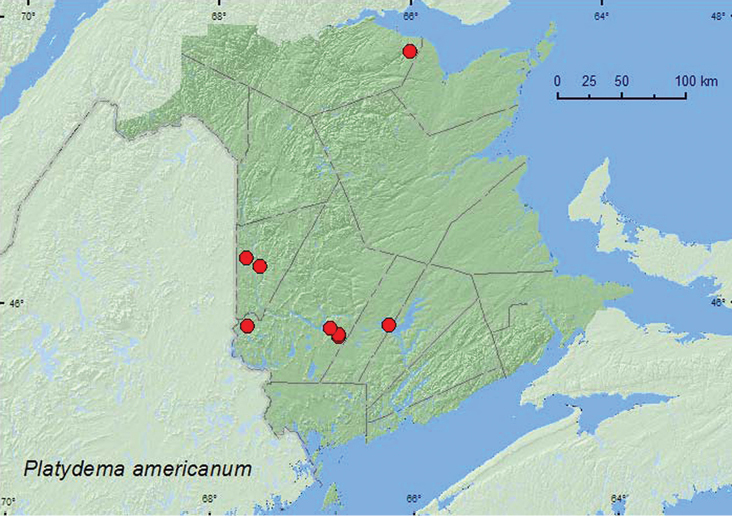
Collection localities in New Brunswick, Canada of *Platydema americanum*.

##### Collection and habitat data.

Most adults from New Brunswick were collected from dried polypore fungi, partially dried *Pleurotus* sp., or other polypore fungi on logs or standing dead trees in mixed and hardwood (silver maple, sugar maple, and beech) forests. One individual was collected from under bark of a conifer stump in late November. This was probably an overwintering site. Adults were collected during May, June, July, August, September, and November.

##### Distribution in Canada and Alaska. 

BC, AB, MB, ON, QC, NB, NS (Bousquet and Campbell 1991; Majka et al. 2008). *Platydema americanum* Laporte and Brullé was reported from New Brunswick in Bousquet and Campbell (1991). However, no voucher specimens could be located to support this record, but Majka et al. (2008) provisionally retained the species on the New Brunswick faunal list. The above records confirm the presence of this species in New Brunswick. This species was reported by Majka et al. (2008) from one locality in Nova Scotia.

#### Platydema excavatum

(Say, 1824)

http://species-id.net/wiki/Platydema_excavatum

##### Remarks.

The specimen of *Platydema excavatum* reported in Majka et al. (2008) was misidentified by C.G. Majka and was a specimen of *Platydema teleops* Triplehorn (collected by R. P. Webster on 5 June 2003, Charters Settlement, N.B., not 3 June 2003 as reported in Majka et al. 2008) (see below). In view of this, *Platydema excavatum* is removed from the faunal list of New Brunswick.

#### Platydema teleops

Triplehorn, 1965

http://species-id.net/wiki/Platydema_teleops

Map 13

##### Material examined.

**New Brunswick, Queens Co.**, Cranberry Lake P.N.A., 46.1125°N, 65.6075°W, 24.IV–5.V.2009, 5–13.V.2009, 21–27.V.2009, R. Webster & M.-A. Giguère, old red oak forest, Lindgren funnel traps (7, AFC, RWC); same locality data and forest type, 3–13.V.2011, 13–25.V.2011, 7–22.VI.2011, 29.VI–7.VII.2011, M. Roy & V. Webster, Lindgren funnel traps (10, AFC, NBM, RWC). **York Co.**, Charters Settlement, 45.8428°N, 66.7279°W, 5.VI.2003, R. P. Webster, regenerating mixed forest, beating foliage (1, RWC); same locality and collector but 45.8395°N, 66.7391°W, 19.V.2007, mixed forest, under bark of large *Populus* sp. log (1, RWC); Canterbury, trail to Browns Mountain Fen, 45.9033°N, 67.6260°W, 2.V.2005, R. Webster & M.-A. Giguère, mixed forest with cedar, margin of vernal pond in moist leaf litter (1, NBM); 15 km W of Tracy off Rt. 645, 45.6848°N, 66.8821°W, 18.V–2.VI.2010, R. Webster & C. MacKay, old red pine forest, Lindgren funnel traps (2, AFC).

**Map 13:**
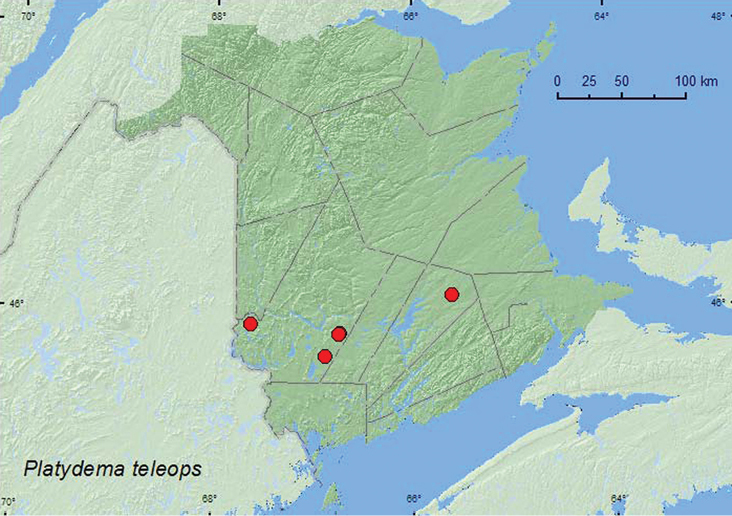
**.** Collection localities in New Brunswick, Canada of *Platydema teleops*.

##### Collection and habitat data.

In New Brunswick, this species was found in red oak, red pine, and mixed forests. Adults were collected from under bark of a *Populus* sp. log, sifted from moist leaf litter on a vernal pond margin, and beaten from foliage. Most adults were captured in Lindgren funnel traps. Adults were collected during April, May, June, and July (most during May).

##### Distribution in Canada and Alaska. 

ON, QC, **NB**, NS (Bousquet and Campbell 1991; Majka et al. 2008).

### Tribe Hypophlaeini Billberg, 1820

#### Corticeus praetermissus

(Fall, 1926)

http://species-id.net/wiki/Corticeus_praetermissus

Map 14

##### Material examined.

**New Brunswick, York Co.,** Charters Settlement, 45.8188°N, 66.7460°W, 16.IV.2005, R. P. Webster, clear-cut, under bark of white pine log (1, RWC); same locality and collector but 45.8286°N, 66.7365°W, 6.VI.2007, mature red spruce and red maple forest, under bark of red spruce infested with bark beetles (1, RWC); 15 km W of Tracy off Rt. 645, 45.6845°N, 66.8807°W, 13.V.2009, R. P. Webster, old red pine forest, under bark scales of recently fallen red pine (1, RWC).

**Map 14.**
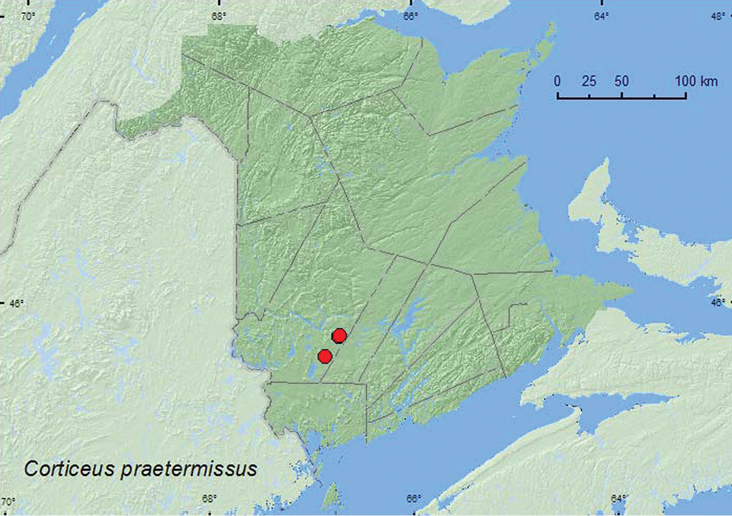
Collection localities in New Brunswick, Canada of *Corticeus praetermissus*.

##### Collection and habitat data.

In New Brunswick, adults were collected under bark of white pine (*Pinus strobus* L.), under bark scales of recently fallen red pine and under bark of a red spruce log infested with bark beetles (*Dendroctonus rufipennis* (Kirby)). Majka et al. (2008) reported this species from similar habitats in Nova Scotia. Adults were collected during April, May, and June.

##### Distribution in Canada and Alaska.

AK, YK, NT, BC, AB, SK, MB, ON, QC, **NB**, NS (Bousquet and Campbell 1991; Majka et al. 2008).

### Subfamily Stenochiinae Kirby, 1837

**Tribe Cnodalonini Oken, 1843**

#### Xylopinus aenescens

LeConte, 1866**

http://species-id.net/wiki/Xylopinus_aenescens

Map 15

##### Material examined.

**New Brunswick, Queens Co.**, Grand Lake Meadows P.N.A., 45.8227°N, 66.1209°W, 19.VII–5.VIII.2011, M. Roy & V. Webster, silver maple swamp and seasonally flooded marsh, Lindgren funnel trap in forest canopy (1, RWC).

**Map 15.**
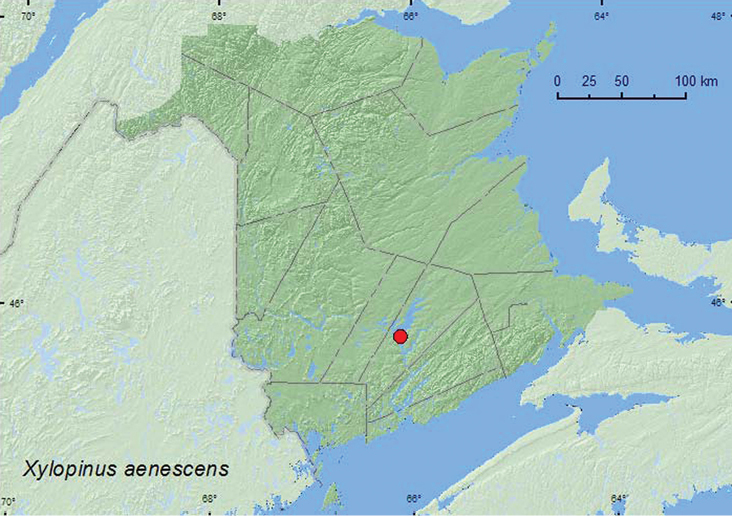
Collection localities in New Brunswick, Canada of *Xylopinus aenescens*.

##### Collection and habitat data.

The New Brunswick specimen was captured between 19 July and 5 August in a Lindgren funnel trap deployed in an old silver maple swamp.

##### Distribution in Canada and Alaska.

QC, **NB** (Bousquet 1991).

#### Xylopinus saperioides

(Olivier, 1795)

http://species-id.net/wiki/Xylopinus_saperioides

Fig. 6 Map 16

##### Material examined.

**New Brunswick, Queens Co.**, Grand Lake near Scotchtown, 45.8762°N, 66.1816°W, 9.VII.2006, R. P. Webster, oak & maple forest, on trunk of large dead standing red oak (collected at night using headlamp) (5, RWC); Grand Lake Meadows P.N.A., 45.8227°N, 66.1209°W, 19.VII–5.VIII.2011, 5–17.VIII.2011, M. Roy & V. Webster, silver maple swamp and seasonally flooded marsh, Lindgren funnel traps in forest canopy (7, AFC, NBM, RWC). **Sunbury Co.**, Burton, near Sunpoke Lake, 45.7763°N, 66.5550°W, 20.VII.2006, R. P. Webster, (red) oak forest, under loose bark of oak (1, RWC); same locality but 45.7658°N, 66.5546°W, red oak & red maple forest, on trunk of dying *Quercus rubra* (collected at night using headlamp) (3, RWC).

**Map 16.**
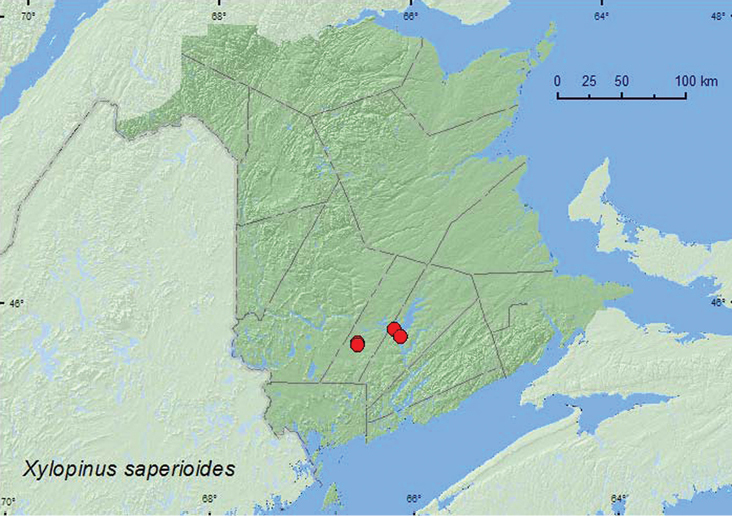
Collection localities in New Brunswick, Canada of *Xylopinus saperdioides*.

##### Collection and habitat data.

Adults of this species were collected in red oak, and red oak and red maple forests, and a silver maple swamp. Many individuals were collected at night from the trunks of dead or dying red oak trees, usually on areas of the trunk without bark. One individual was collected from under loose bark of an oak during the day. Other individuals were captured in Lindgren funnel traps deployed in the canopy of silver maples. Adults were collected during July and August.

##### Distribution in Canada and Alaska.

ON, QC, **NB**, NS (Bousquet and Campbell 1991; Majka et al. 2008). This species was first reported from the Maritime provinces by Majka et al. (2008) based on a specimen from Nova Scotia, Queens Co. Kejimkujik National Park (D.C. Ferguson).

### Family Zopheridae Solier, 1834

**Subfamily Colydiinae Billberg, 1820**

**Tribe Synchitini Erichson, 1845**

#### Bitoma crenata

Fabricius, 1775**

http://species-id.net/wiki/Bitoma_crenata

Map 17

##### Material examined.

**New Brunswick, York Co.**, 15 km W of Tracy off Rt. 645, 45.6845°N, 66.8807°W, 27.VIII.2008, R. P. Webster, old red pine forest, under bark of *Populus* sp. log covered with dried polypore fungus (11, NBM, RWC).

**Map 17.**
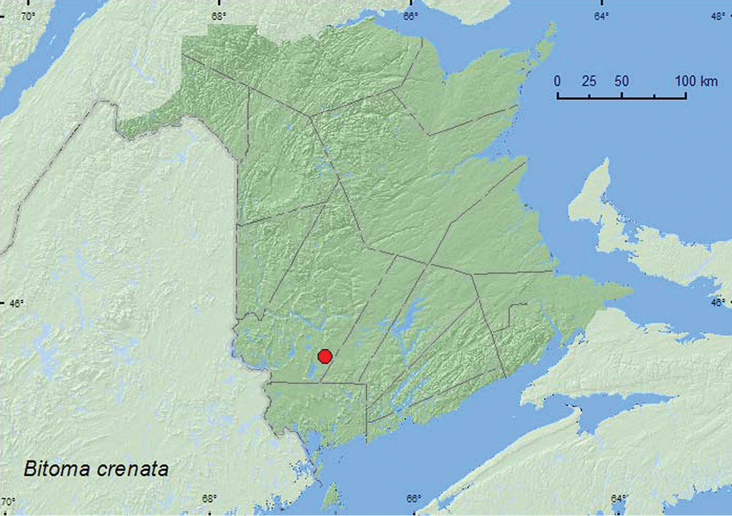
Collection localities in New Brunswick, Canada of *Bitoma crenata*.

##### Collection and habitat data.

New Brunswick specimens of this adventive species were collected from under bark of a *Populus* sp. log (sun-exposed) covered with polypore fungi. This species was reported from similar habitats by Westcott et al. (2006).

##### Distribution in Canada and Alaska.

ON, QC, **NB** (Bousquet 1991).

#### Synchita fuliginosa

Melsheimer, 1846

http://species-id.net/wiki/Synchita_fuliginosa

Map 18

##### Material examined.

**New Brunswick, Carleton Co.**, Jackson Falls, Bell Forest, 46.2200°N, 67.7231°W, 8.VII.2008, R. P. Webster, mature hardwood forest, u.v. light (1, RWC). **Queens Co.**, Cranberry Lake P.N.A. 46.1125°N, 65.6075°W, 1–10.VII.2009, 10–15.VII.2009, 21–18.VII.2009, R. Webster & M.-A. Giguère, old red oak forest, Lindgren funnel traps (3, RWC);Grand Lake Meadows P.N.A., 45.8227°N, 66.1209°W, 31.V–15.VI.2010, 15–29.VI.2010, 29.VI–12.VII.2010, R. Webster, C. MacKay, M. Laity, & R. Johns, old silver maple forest with green ash and seasonally flooded marsh, Lindgren funnel traps (7, AFC). **Victoria Co.**, Riley Brook, (no collector given) reared from bolts of *Ulmus americana* collected on 31.VI.1972, adults emerged January and February, 1973 (4, AFC). **York Co.**, 15 km W of Tracy off Rt. 645, 45.6848°N, 66.8821°W, 7–14.VII.2009, 14–20.VII.2009, 20–29.VII.2009, 29.VII–4.VIII.2009, R. Webster & M.-A. Giguère, old red pine forest, Lindgren funnel traps (5, RWC).

**Map 18.**
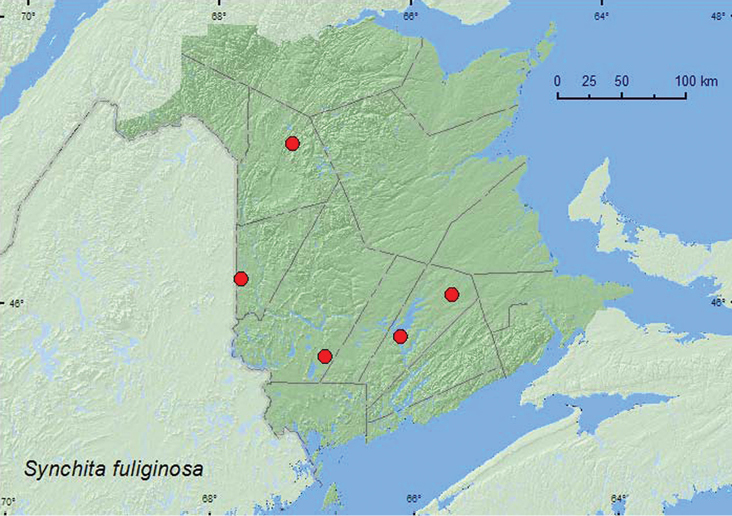
Collection localities in New Brunswick, Canada of *Synchita fuliginosa*.

##### Collection and habitat data.

Most (16) adults from New Brunswick were captured in Lindgren funnel traps deployed in a mature hardwood forest, an old red oak forest, an old silver maple swamp, and an old red pine forest. Individuals with more specific bionomic data were reared from American elm (*Ulmus americana* L.) bolts and taken at an ultraviolet light. Elsewhere, *Synchita fuliginosa* have been found under bark of a variety of hardwood species or collected at light (Stephan 1989).

##### Distribution in Canada and Alaska.

ON, QC, **NB**, NS (Bousquet 1991; Majka et al. 2006).

## Supplementary Material

XML Treatment for Paratenetus punctatus

XML Treatment for Eleates depressus

XML Treatment for Neatus tenebrioides

XML Treatment for Tribolium castaneum

XML Treatment for Pseudocistela brevis

XML Treatment for Isomira sericea

XML Treatment for Mycetochara analis

XML Treatment for Mycetochara bicolor

XML Treatment for Mycetochara binotata

XML Treatment for Mycetochara foveata

XML Treatment for Neomida bicornis

XML Treatment for Platydema americanum

XML Treatment for Platydema excavatum

XML Treatment for Platydema teleops

XML Treatment for Corticeus praetermissus

XML Treatment for Xylopinus aenescens

XML Treatment for Xylopinus saperioides

XML Treatment for Bitoma crenata

XML Treatment for Synchita fuliginosa
